# Supplementary material for: Genome-Wide Identification and Expression Analysis of SOS Response Genes in Salmonella enterica Serovar Typhimurium
Source: Cells. 2021 Apr 19;10(4):943. doi: 10.3390/cells10040943 (PMC8072944; doi:10.3390/cells10040943)
Supplement: Supplementary file 1 [file cells-10-00943-s001.zip › Table S2.pdf]

Genome-wide identification and expression analysis of SOS response genes in *Salmonella enterica* serovar Typhimurium

Ángela Mérida-Floriano and Josep Casadesús

Table S2. Strains used in this study.

**Strain**	**Genotype**
ATCC 14028	Wild type
ST4/74	Wild type
SV4231 [b]	*recF522*::Tn5
SV4212 [b]	*recD543*::Tn10
SV10037/42	14028 [SNP4/74]-*yebG::gfp*
SV10043/44	4/74 [SNP14028]-*yebG::gfp*
SV9012	*yebG*::*gfp*
SV9063	*yebG*::*gfp lexA*(Ind⁻)
SV9407	*umuC*::*gfp*
SV9408	*uvrD*::*gfp*
SV9409	*ruvA*::*gfp*
SV9410	*recA*::*gfp*
SV9453	*recN*::*gfp*
SV9454	STM14_3210::*gfp*
SV9455	STM14_1156::*gfp*
SV9462	*dinI*::*gfp*
SV9463	*yigN*::*gfp*
SV9464	*dinP*::*gfp*
SV9465	*nlhH*::*gfp*
SV9467	*recA*::*gfp lexA*(Ind⁻)
SV9468	*uvrD*::*gfp lexA*(Ind⁻)
SV9469	*umuC*::*gfp lexA*(Ind⁻)
SV9470	*ruvA*::*gfp lexA*(Ind⁻)
SV9471	*recN*::*gfp lexA*(Ind⁻)
SV9472	*dinI*::*gfp lexA*(Ind⁻)
SV9473	*yigN*::*gfp lexA*(Ind⁻)
SV9474	*dinP*::*gfp lexA*(Ind⁻)
SV9475	STM14_3210::*gfp lexA*(Ind⁻)
SV9476	*nlhH*:*gfp lexA*(Ind⁻)
SV9477	STM14_1156::*gfp lexA*(Ind⁻)
SV9479	*sulA*::*gfp*
SV9480	*sulA*::*gfp lexA*(Ind⁻)
SV9546	STM14_1156::*gfp* Δ*recA*::*Cm*
SV9618	Δ*recA::Cm*
SV9635	*yebG*::*gfp* Δ*recA*::*Cm*
SV9636	*sulA*::*gfp* Δ*recA*::*Cm*
SV9637	*dinI*::*gfp* Δ*recA*::*Cm*

SV9638	*uvrD*::*gfp* Δ*recA*::*Cm*
SV9639	STM14_3210::*gfp* Δ*recA*::*Cm*
SV9671	*ssb*::*gfp*
SV9673	STM14_3214::*gfp*
SV9674	*lexA*::*gfp*
SV9675	IstR-1,2::*gfp*
SV9676	*polB*::*gfp*
SV9677	*yqaB*::*gfp*
SV9678	*uvrA*::*gfp*
SV9679	*lexA*::*gfp* Δ*recA*::*Cm*
SV9680	IstR-1,2::*gfp* *lexA*(Ind^{-})
SV9681	*polB*::*gfp* *lexA*(Ind^{-})
SV9682	*yqaB*::*gfp* *lexA*(Ind^{-})
SV9683	*uvrA*::*gfp* *lexA*(Ind^{-})
SV9685	*ssb*::*gfp* *lexA*(Ind^{-})
SV9687	STM14_3214::*gfp* *lexA*(Ind^{-})
SV9697	*yejL*::*gfp*
SV9698	*yejL*::*gfp* *lexA*(Ind^{-})
SV9708	*msgA*::*gfp*
SV9709	*msgA*::*gfp* *lexA*(Ind^{-})
SV9710	*corE*::*gfp*
SV9711	*corE*::*gfp* *lexA*(Ind^{-})
SV9712	*sbmC*::*gfp*
SV9713	*sbmC*::*gfp* *lexA*(Ind^{-})
SV9719	*uvrB*::*gfp*
SV9722	STM14_1432::*gfp*
SV9725	*uvrB*::*gfp* *lexA*(Ind^{-})
SV9728	STM14_1432::*gfp* *lexA*(Ind^{-})
SV9731	ST4/74 *yebG::gfp*
SV9733	*yqaB*::*gfp* Δ*recA*::*Cm*
SV9736	*msgA*::*gfp* Δ*recA*::*Cm*
SV9737	*yejL*::*gfp* Δ*recA*::*Cm*
SV9738	STM14_3214::*gfp* Δ*recA*::*Cm*
SV9740	*ssb*::*gfp* Δ*recA*::*Cm*
SV9741	IstR-1,2::*gfp* Δ*recA*::*Cm*
SV9742	*nlhH*::*gfp* Δ*recA*::*Cm*
SV9743	*polB*::*gfp* Δ*recA*::*Cm*
SV9747	*sbmC*::*gfp* Δ*recA*::*Cm*
SV9748	*ruvA*::*gfp* Δ*recA*::*Cm*
SV9749	*umuC*::*gfp* Δ*recA*::*Cm*
SV9750	*uvrA*::*gfp* Δ*recA*::*Cm*
SV9752	*uvrB*::*gfp* Δ*recA*::*Cm*
SV9753	*corE*::gfp Δ*recA*::*Cm*
SV9754	*dinP*::*gfp* Δ*recA*::*Cm*

SV9755	*yigN*::*gfp* Δ*recA*::*Cm*
SV9756	*recN*::*gfp* Δ*recA*::*Cm*
SV9757	STM14_1432::*gfp* Δ*recA*::*Cm*
SV10122	*sulA*::*gfp* Δ*recF522*::Tn5
SV10037/42	[SNP4/74]-*yebG::gfp*
SV10043/44 c	[SNP14028]-*yebG::gfp*
SV10123	*sulA*::*gfp* Δ*recD543*::Tn10

[a] Allele originally described by Bunny et al. 2002 [18]

[b] Alleles originally described by Cano et al. 2002 [20]

[c] 4/74 derivative
