# Supplementary material for: Genome-Wide Identification and Expression Analysis of SOS Response Genes in Salmonella enterica Serovar Typhimurium
Source: Cells. 2021 Apr 19;10(4):943. doi: 10.3390/cells10040943 (PMC8072944; doi:10.3390/cells10040943)
Supplement: Supplementary file 1 [file cells-10-00943-s001.zip › Table S3.pdf]

Ángela Mérida-Floriano and Josep Casadesús

Table S3. Oligonucleotides used in this study.

**Name**	**Sequence (5’ → 3’)**
yebG-GFP-F	AAAGGACGCCGCGAGTAAAACGAAAAAGCAAGCCGCCTGATAAGAAGGAGATATACATATGAG
yebG-GFP-Km-R	GAAAAGCATGGTACAAAACGCCGGGAGCGCAAGTGTGGGTTTCAGGCGTAGCACCAGGCG
yebG-E1	TACCTGCGCGCAGTATATAC
yebG-E2	CCTTAGCACAATGATTCAGG
sulA-E1	ATAGAGTTGATCTTTCATCG
sulA-E2	CTTCTGACAAGCGTTCTCGC
recA-P1	GGCGACGACATACGCGCTACAAACCCTGTGGCAACAATTTGTGTAGGCTGGAGCTGCTTC
recA-P2	ATGGCGGCTTCGTTTTGCCCGCCCCACCATCACCTGATGACATATGAATATCCTCCTTAG
recA-E1	TGGAGAAGGGATTACGCGTC
recA-E2	TTACGCCGTAATTCCTGCTC
recN-GFP-F	TAATACGCTGGCAAACGCAAAAGAACTGCTGGCGGCGTAATAAGAAGGAGATATACATATGAG
recN-GFP-Km-R	GTTTTTGTTGTGAACTCAGACCTTCGGGAGATAAAAAAGTTTCAGGCGTAGCACCAGGCG
recN-E1	AAGATGTGCTGATTCGCCGC
recN-E2	CAGTCAGCGTTTTACAGCGC
sulA-GFP-F	GCATTCCGGGCTAAAAATTCACTCTAATTTGTATCATTAATAAGAAGGAGATATACATATGAG
sulA-GFP-Km-R	GTTCGCGTAAAAAAAAGTCCAGGATAAATCCTAATTTTACTTCAGGCGTAGCACCAGGCG
STM13_3210-GFP-F	GCAGGAAACGTGGGAAAGCGCTGACGACTGGTTTTATTAATAAGAAGGAGATATACATATGAG
STM14_3210-GFP-Km-R	GCGGTATTCTGAAAAATGCGCGCCAGCATTAAGCGCAATGTTCAGGCGTAGCACCAGGCG
STM14_3210-E1	AGCCGAATGAAATTGCAGTC
STM14_3210-E2	GAAAGCTAAACACCACGCAG
dinI-GFP-F	GACCTGGGAGAGCGCTGATGACTGGTTCATCAATGAATAATAAGAAGGAGATATACATATGAG
dinI-GFP-Km-R	CGGGGCGACCCGGCAAACATACATCACTGCATATCATTTTTTCAGGCGTAGCACCAGGCG
dinI-E1	GATGAACAACAGATACCGGG
dinI-E2	TATGAACAATAAAGCGGCAG
nlhH-GFP-F	CTGGCGTGTTGTAAGTCCATCAACAGGCAAAATGACCTAATAAGAAGGAGATATACATATGAG

nlhH-GFP-Km-R	CAACGTTATCCAGTTTTCAAGTTGAAAACAAAATTGAATTTTCAGGCGTAGCACCAGGCG
nlhH-E1	CCAGAATCATTATCCCCTCG
nlhH-E2	AACGGTTTTCGGCTGATAAC
STM14_1156-GFP-F	GCAGGAAACGTGGGAAAGCGCTGACGACTGGTTTTATTAATAAGAAGGAGATATACATATGAG
STM14_1156-GFP-Km-R	GCGATATTCTGAAAAATGCGCGCCAGCATTAAGCGCAATGTTCAGGCGTAGCACCAGGCG
STM14_1156-E1	TTGAATGAATGTGAACCCGC
STM14_1156-E2	GATAGGACTCCCTCTGCCAC
dinP-GFP-F	CGACCCTCAACTGGAACGACAGTTGGTGTTAGGGTTATAATAAGAAGGAGATATACATATGAG
dinP-GFP-Km-R	TGGCGCAATCGTAATAGTGATAAAATATTAATGGCCTGACTTCAGGCGTAGCACCAGGCG
dinP-E1	ACGTTTACAATTTTCCCGCC
dinP-E2	TGAGAATGAGAAAACGCGGG
dinJ-GFP-F	CAAAGATGCTGATGATTTGTTTGACCAGTTAGGGATCTGATAAGAAGGAGATATACATATGAG
dinJ-GFP-Km-R	TGGAACTGTCCCGAATATTCAATTTCCCTTTGCCCCATAGTTCAGGCGTAGCACCAGGCG
dinJ-E1	AGCATTGCAAGGCAAACATC
dinJ-E2	TGCCGACATCCTTATGACGC
STM14_3214-GFP-F	CCACTGGAACAGCCCGGATGCATGGAGGGATTTCCTATGATAAGAAGGAGATATACATATGAG
STM14_3214-GFP-Km-R	GCGCCATCCCGATTGTTTATTGCGCGGTAGAGTTCAGGTTTTCAGGCGTAGCACCAGGCG
STM14_3214-I1	CTCGGAGCTGACCGGCATTC
STM14_3214-E2	GCTTCAGTTGCATGAACAGC
umuC-GFP-F	GTACACCACAAGATACTCTGATCTACTGCGTGTTAAGTAATAAGAAGGAGATATACATATGAG
umuC-GFP-Km-R	GGGATGATTTGGCAACCATCTCAGGCATTGATCGCACAAGTTCAGGCGTAGCACCAGGCG
umuC-E1	ACTTCAATGTCGTGCTGGAG
umuC-E2	ATACTCTTTTTATTCATGCC
STM14_1432-GFP-F	CCTGAATAACACTGACTGGATTTACGGAGTGGAGCTATGATAAGAAGGAGATATACATATGAG
STM14_1432-GFP-Km-R	TGCTCACGGTCAAAATTAACCATCTGCGCAGCAATGTTTTTTCAGGCGTAGCACCAGGCG
STM14_1432-E1	GAACACCAACGGCTTTCTGG
STM14_1432-E2	CTGTTCCGGCATGTTGTTGG
recA-GFP-F	CGATAGCGAAGGCGTTGCAGAAACCAACGAAGATTTTTAATAAGAAGGAGATATACATATGAG
recA-GFP-Km-R	ATGGCGGCTTCGTTTTGCCCGCCCCACCATCACCTGATGATTCAGGCGTAGCACCAGGCG
yigN-GFP-F	TTATCCCAATGATGAACGGGTGAAGCAGCAGTTAAGTTAATAAGAAGGAGATATACATATGAG
yigN-GFP-Km-R	TTGCCCTACTCTGCGCCAGGACTAAACTACTCCGTATGGGTTCAGGCGTAGCACCAGGCG
yigN-E1	CCTCTCTTTTGCAAAGGCCG

yigN-E2	CCACCATGTCAGCTTTCTGC
uvrD-GFP-F	ATGGCTGGTTGCCGCGTATGCGAAGCTGGAAACGGTTTGATAAGAAGGAGATATACATATGAG
uvrD-GFP-Km-R	AGGCCGGATAAGACGCGCCATCCGGCACTATGAGAGTCGGTTCAGGCGTAGCACCAGGCG
uvrD-I1	GTATGAGCAAGAAAAAGGCG
uvrD-E2	GCCAGCAAAAATTTGCCGTC
IstR-1,2-GFP-F	CATTACGCTGTAAGAACCTCGCTTCGGCGAGGTTTTTTGTTAAGAAGGAGATATACATATGAG
IstR-1,2-GFP-Km-R	TCGCCGGGATTGATGATCACGCATTTCACAATGCCGGAAATTCAGGCGTAGCACCAGGCG
IstR-1,2-E1	CCCGTTTCAGCCAGGACTTC
IstR-1,2-E2	ACACGCTTTGAGTATAGGCG
ssb-GFP-F	CGAACCGCCAATGGATTTTGACGACGACATTCCGTTCTGATAAGAAGGAGATATACATATGAG
ssb-GFP-Km-R	AGCGAAAATGCAAATAACGCTTTGAGTTTTTTACCAAAAATTCAGGCGTAGCACCAGGCG
ssb-E1	GTAGAGACTCGGATGCCTGG
ssb-E2	CACCAGGCATTTCAGCAGAC
polB-GFP-F	TTTTGCTACACTACTGACCGGGCAACTGGGGTTATTTTGATAAGAAGGAGATATACATATGAG
polB-GFP-Km-R	AGGGCGCTATGGTACTGGAAGGCGGCGCTTTCGTCACGCGTTCAGGCGTAGCACCAGGCG
polB-I1	GGAAATCGGGCACAGGCTGG
polB-E2	GCTTTCTGTATCGCTGATCC
ruvA-GFP-F	TGAAACACTGATTCGCGACGCGTTACGCGCCGCGTTATGATAAGAAGGAGATATACATATGAG
ruvA-GFP-Km-R	CCTGCTGAAATCAGGCGATCTGCTTCTATCATCCTTTACCTTCAGGCGTAGCACCAGGCG
ruvA-E1	ACCGATTCGAAGGGAAGGCC
ruvA-E2	AGAGTCGTTTTACCTAACCC
uvrB-GFP-F	GTTGCACCAGTTACGCGAGTTGTTTATCGCGGCGTCCTGATAAGAAGGAGATATACATATGAG
uvrB-GFP-Km-R	GAGCCATGGTAACGATGACTCGCTGGCGATCGACACATTGTTCAGGCGTAGCACCAGGCG
uvrB-E1	GATAACCAGCACACTGCTGG
uvrB-E2	CGTTAGTCAAAGCCTGTCAC
sbmC-GFP-F	CTGGGATATTGAAATGTATATTCCGGTACAGCGGAAGTGATAAGAAGGAGATATGAG
sbmC-GFP-Km-R	ACATTATCCGTCCGCTCTTTTCAGCCAGGAAACCAGTCGATTCAGGCGTAGCACCAGGCG
sbmC-E1	ACAAAATGATCACGCTGTCC
sbmC-E2	TTGCGCTCCTTACGGGAATG
msgA-GFP-F	GATGTTTGAAGAGGCTGATATGTGGCTGGTCGCCGAATAATAAGAAGGAGATATACATATGAG
msgA-GFP-Km-R	CTGTTTTCGATCGGACATGTCGCTTTCGCAGGAGGGGACGTTCAGGCGTAGCACCAGGCG
msgA-E1	AACGTCAGGATGACAAAACG

msgA-E2	GAATGTCGGTTCCACATACC
uvrA-GFP-F	GTCGCATACCGCCCGCTTCCTTAAACCTATGCTCAAATAATAAGAAGGAGATATACATATGAG
uvrA-GFP-Km-R	ATGATGCCGCTTCCCGGCGAATCGGAAAGCGGCGTGAAGGTTCAGGCGTAGCACCAGGCG
uvrA-I1	GTGCCGGAGTCTCGCTCGCG
uvrA-E2	GCGCTAAAGCGGTCAGGCAG
yqaB-GFP-F	GGCGGCGGGAATGGATGCCGTGGATGTCCGACTATTGTGATAAGAAGGAGATATACATATGAG
yqaB-GFP-Km-R	AAACTACTGGCAAACAGAGACAGGAGCGACAGTCCGTCACTTCAGGCGTAGCACCAGGCG
yqaB-E1	CTGCCCTGCAATCGCTTTTC
yqaB-E2	CCCCTCCAAGGCTATTACCC
yejL-GFP-F	CGCCTTGCAGTCTTCGATTAGCGAAGACAACGCGCACTAATAAGAAGGAGATATACATATGAG
yejL-GFP-Km-R	AGTTACCATACCTGTCGCTTGTTTCTCCGGAGTCGGCTTTTTCAGGCGTAGCACCAGGCG
yejL-E1	GTCATTTCAGGCGTTATCCG
yejL-E2	TTGAACAGAGCGAACCAGTG
corE-GFP-F	CTATTTTGGTAGCCGCATATTACAGCAATTTGTAAGCTAATAAGAAGGAGATATACATATGAG
corE-GFP-Km-R	GGTGGTGGAGATGTGTTCCAGGGGAAACTCCTTTAAAAGCTTCAGGCGTAGCACCAGGCG
corE-E1	CAAACATTGTCTCTCGCCTG
corE-E2	AAATAGGCCGAGATGACCAC
lexA-GFP-F	GCTGGCGGTAGGCGTCATTCGCAACGGGGAATGGTTGTAGTAAGAAGGAGATATACATATGAG
lexA-GFP-Km-R	CGATTGCCGGATGGCGGCTTACAAGGAGATTAAAAAGAGATTCAGGCGTAGCACCAGGCG
lexA-E1	ACATTTTCGATAAAGGTTCC
lexA-E2	CTGATAAGCGCAGCGCCATC
ΔrecA-GFP-F	ACTATCCGGTTCAATACCAAGTTGCATGACAGGAGTAATATAAGAAGGAGATATACATATGAG
SNP-14028-W85	CCGCCATTTTGCAGCCCCTTATTTACCTGTGTTTATATACACTGGCCGTCGTTTTACAAC
SNP-474-W85	CCGCCATTTTGCAGCCCCTTATTTAGCTGTGTTTATATACACTGGCCGTCGTTTTACAAC
SNP-W86	TTTCGAATAAATTGCTTTTCTTGCTGCCTTGCAATATACTTCGGTCATTGGCATGTTCAA
SNP-yebG-14028	GTATTTAACTTCAACCGCCATTTTGCAGCCCCTTATTTAGCTGTGTTTATATACAGTATATTGCAAGGCAGCAAGAA AAGCAATTTATTC
SNP-yebG-474	GTATTTAACTTCAACCGCCATTTTGCAGCCCCTTATTTACCTGTGTTTATATACAGTATATTGCAAGGCAGCAAGAA AAGCAATTTATTC
