# Supplementary material for: Genome-Wide Identification and Expression Analysis of SOS Response Genes in Salmonella enterica Serovar Typhimurium
Source: Cells. 2021 Apr 19;10(4):943. doi: 10.3390/cells10040943 (PMC8072944; doi:10.3390/cells10040943)
Supplement: Supplementary file 1 [file cells-10-00943-s001.zip › Table S1.pdf]

Genome-wide identification and expression analysis of SOS response genes in *Salmonella enterica* serovar Typhimurium

Ángela Mérida-Floriano and Josep Casadesús

Table S1. SOS response genes identified in *E. coli* and *S. enterica*. Genes highlighted in bold are shared by this study and others.

**Fernandez de Henestrosa *et al*. 2000 (42)** (*E. coli*)	**Wade *et al*. 2005 (9)** (*E. coli*)	**Erill *et al*. 2003 (2)** (*S. enterica*)	**This work** (*S. enterica*)
dinB	***dinB***	***dinB***	***dinB (dinP)***
dinG	***dinG***	***dinG***	***dinG***
dinI	***dinI***	***dinI***	***dinI***
lexA	***lexA***	***lexA***	***lexA***
polB	***polB***	***polB***	***polB***
recA	***recA***	***recA***	***recA***
recN	***recN***	***recN***	***recN***
sbmC	***sbmC***	***sbmC***	***sbmC***
ssb	***ssb***	***ssb***	***Ssb***
sulA	***sulA***	***sulA***	***sulA***
umuDC	***umuDC***	***umuDC***	***umuDC***
uvrA	***uvrA***	***uvrA***	***uvrA***
uvrB	***uvrB***	***uvrB***	***uvrB***
uvrD	***uvrD***	***uvrD***	***uvrD***
ydjM	***ydjM***	***ydjM***	***ydjM***
yebG	***yebG***	***yebG***	***yebG***
yigN	***yigN***	***yigN***	***yigN***
brnQ	***ydjQ***	***ydjQ***	***ydjQ***
creA	*cadB*	***msgA***	***msgA***
dinQ	*clpX*	**STM14_1156**	**STM14_1156**
dinS	*dinD*	**STM14_3210**	**STM14_3210**
dinJ	*dinQ*	*ftsK*	***dinJ***
ecpD	*fadE*	*ftsY*	*corE*
ftsK	*ftsK*	*mfd*	*cysP*
hofQ	*hokE*	*minC*	*deoD*
hokE	*idi*	*molR*	*frsA*
ilbL	*intD*	*recG*	*gudD*
ilvD	*malI*	*ruvA*	*higB*
metE	*minC*	STM0925	*hupA*
metR	*molR*	STM14_1198	IstR-1,2*

minC	*otsB*	STM2727	*mutH*
molR	*polA*	*ybfE*	*ndh*
mug	*ptrA*	*yjiW*	*nlhH*
pcsA / dinD	*ruvA*		*rcnB*
pshM	*ybbK*		*slyX*
rfaJ	*ybeR*		STM14_1432
rob	*ybeX*		STM14_1439
ruvAB	*ybjK*		STM14_2473
tyrS	*ydeQ*		STM14_3214
xylE	*ydfJ*		STM14_5518
yafL	*ydjF*		*thiM*
ybfE	*yebN*		*tisB* *
ybiA	*yfaX*		*yacA*
ybiT	*ygcK*		*yejK*
ycbU	*yjiW*		*yejL*
ycgJ	*ynaE*		*yhjE*
ycgL	*yoaC*		*yqaB*
yciG	*ysdA*		
ydbH			
ydeJ			
ydJjQ			
yecS			
yfiE			
yfiK			
ygiS			
yhiX			
yiaO			
yjgN			
yjiW			
ysdAB			

* LexA-mediated control of *istR-1,2* and *tisB* was also described by Vogel et al (45).
