# Supplementary material for: Genome-Wide Identification and Expression Analysis of SOS Response Genes in Salmonella enterica Serovar Typhimurium
Source: Cells. 2021 Apr 19;10(4):943. doi: 10.3390/cells10040943 (PMC8072944; doi:10.3390/cells10040943)
Supplement: Supplementary file 1 [file cells-10-00943-s001.zip › Figure S1.pdf]

Genome-wide identification and expression analysis of SOS response genes in *Salmonella enterica* serovar Typhimurium

Ángela Mérida-Floriano and Josep Casadesús

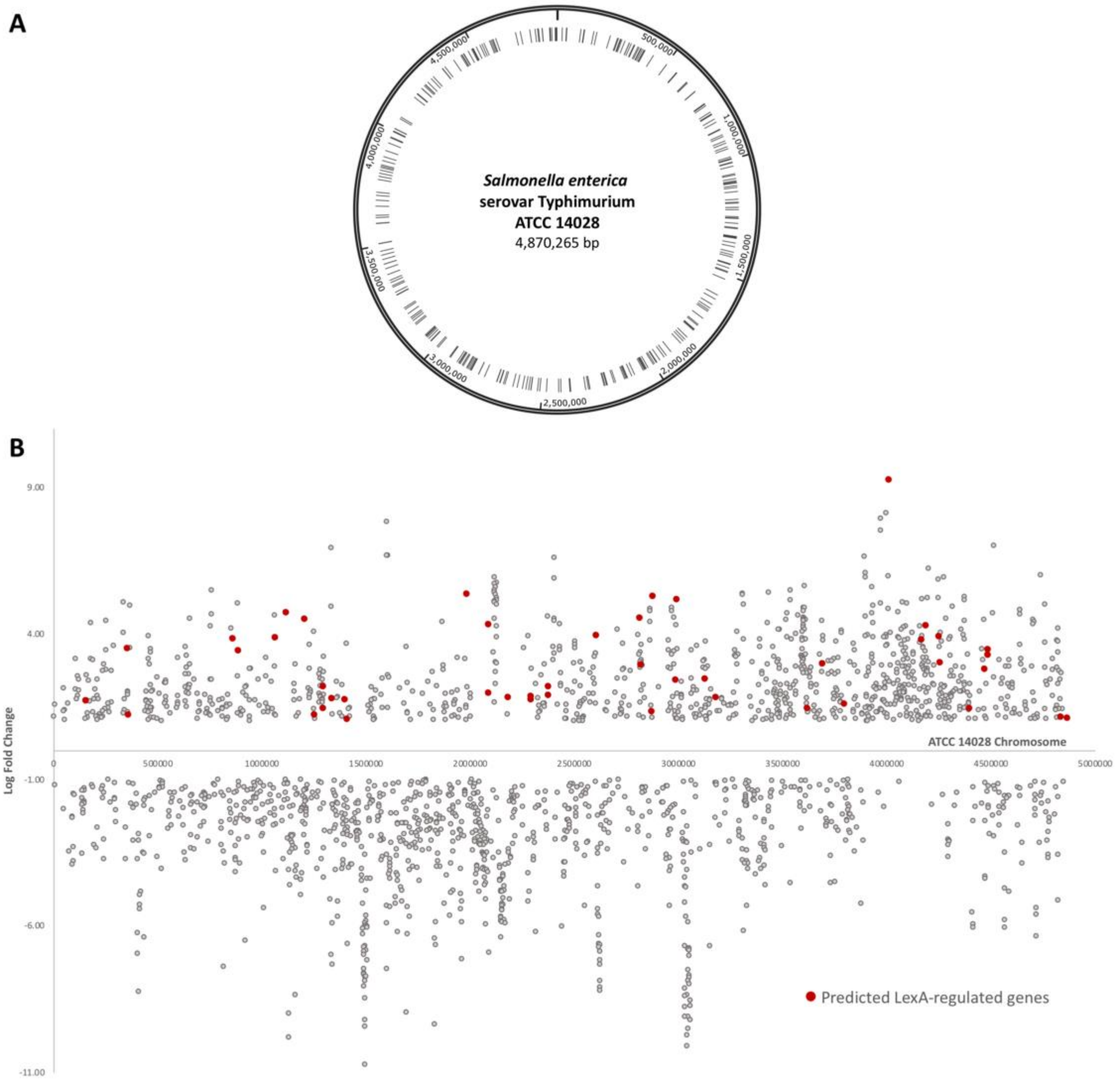

Figure S1. Identification of SOS response genes. **A.** Location of the 318 putative LexA boxes identified by bioinformatic analysis in the chromosome of *S. enterica* ATCC 14028. **B.** Chromosomal location of genes differentially expressed in the presence of 8,5 µg/ml of nalidixic acid. In red: genes upregulated by nalidixic acid that present a SOS box in their promoter region (listed in Table 1).
