# Supplementary material for: Genome-Wide Identification and Expression Analysis of SOS Response Genes in Salmonella enterica Serovar Typhimurium
Source: Cells. 2021 Apr 19;10(4):943. doi: 10.3390/cells10040943 (PMC8072944; doi:10.3390/cells10040943)
Supplement: Supplementary file 1 [file cells-10-00943-s001.zip › Figure S3.pdf]

Ángela Mérida-Floriano and Josep Casadesús

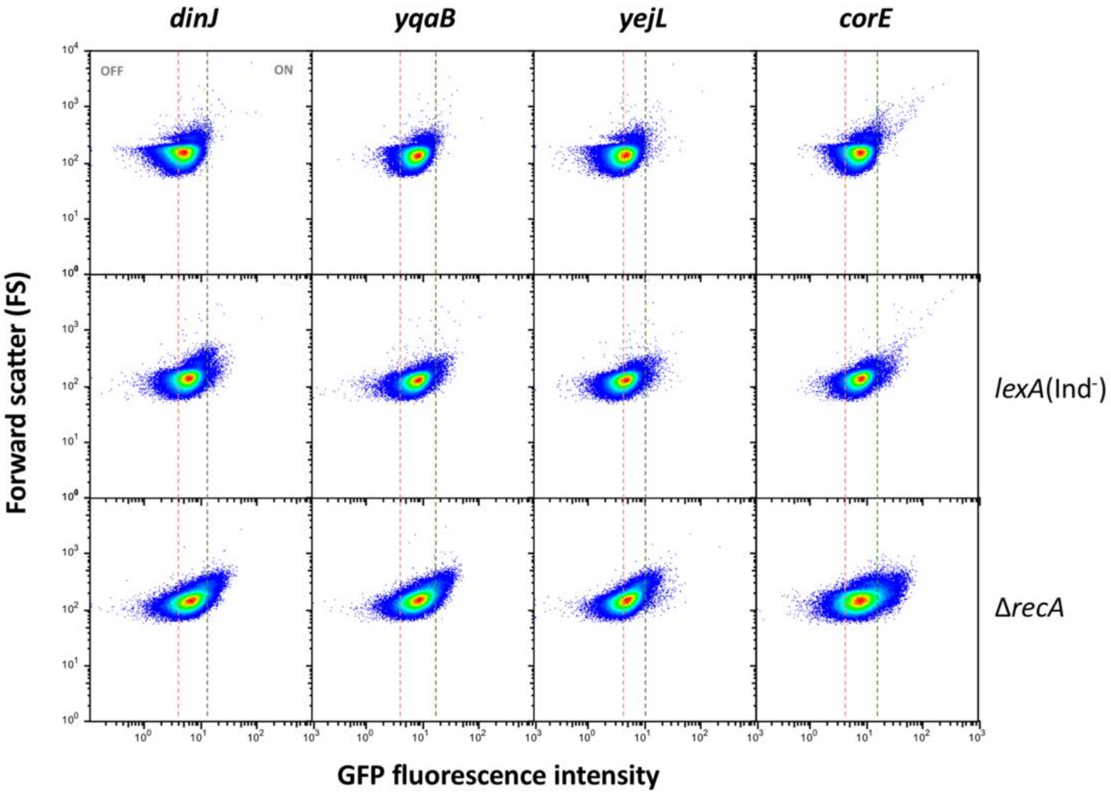

Figure S3. Expression patterns of miscellaneous loci**.** The expression of genes in this group is heterogeneous but a common trait is the presence of a subpopulation that only appears in a LexA(Ind—) background.
