# Supplementary material for: Genome-Wide Identification and Expression Analysis of SOS Response Genes in Salmonella enterica Serovar Typhimurium
Source: Cells. 2021 Apr 19;10(4):943. doi: 10.3390/cells10040943 (PMC8072944; doi:10.3390/cells10040943)
Supplement: Supplementary file 1 [file cells-10-00943-s001.zip › Figure S4.pdf]

Genome-wide identification and expression analysis of SOS response genes in *Salmonella enterica* serovar Typhimurium

Ángela Mérida-Floriano and Josep Casadesús

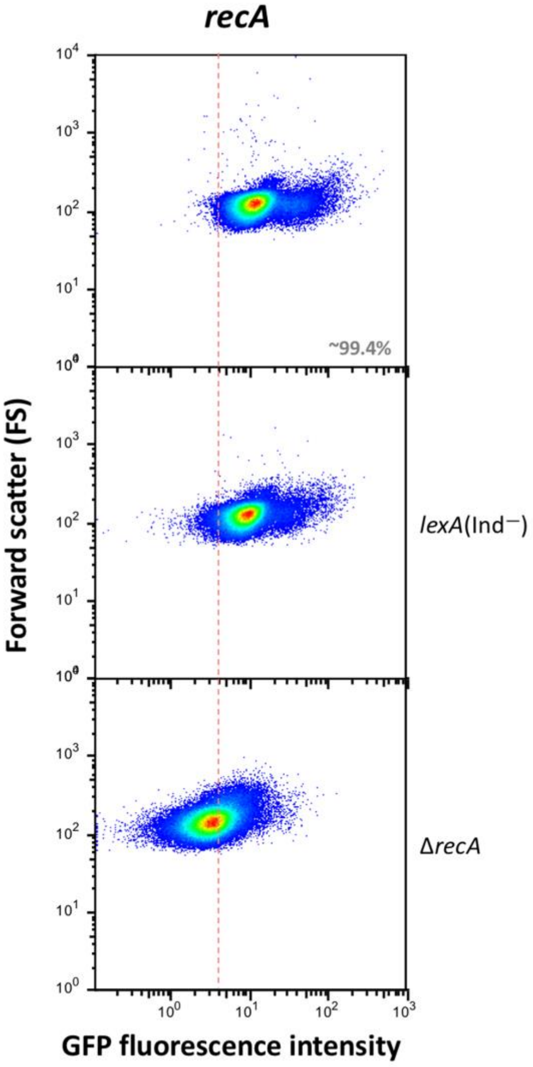

Figure S4. Expression pattern of *recA*. Nearly all cells in a non-induced culture express *recA*. A subpopulation of ~5.6% cells presents higher levels of GFP fluorescence, indicating that *recA* is expressed at higher rates in these cells. In a LexA(Ind⁻) background, this subpopulation decreases to ~3.5%, but does not disappear completely, suggesting that LexA is not entirely responsible for the heterogeneity of *recA* expression. In the absence of RecA, the activity of the *recA* promoter decreases to 32% but the expression remains heterogeneous.
