# Supplementary material for: Genome-Wide Identification and Expression Analysis of SOS Response Genes in Salmonella enterica Serovar Typhimurium
Source: Cells. 2021 Apr 19;10(4):943. doi: 10.3390/cells10040943 (PMC8072944; doi:10.3390/cells10040943)
Supplement: Supplementary file 1 [file cells-10-00943-s001.zip › Figure S5.pdf]

Genome-wide identification and expression analysis of SOS response genes in *Salmonella enterica* serovar Typhimurium

Ángela Mérida-Floriano and Josep Casadesús

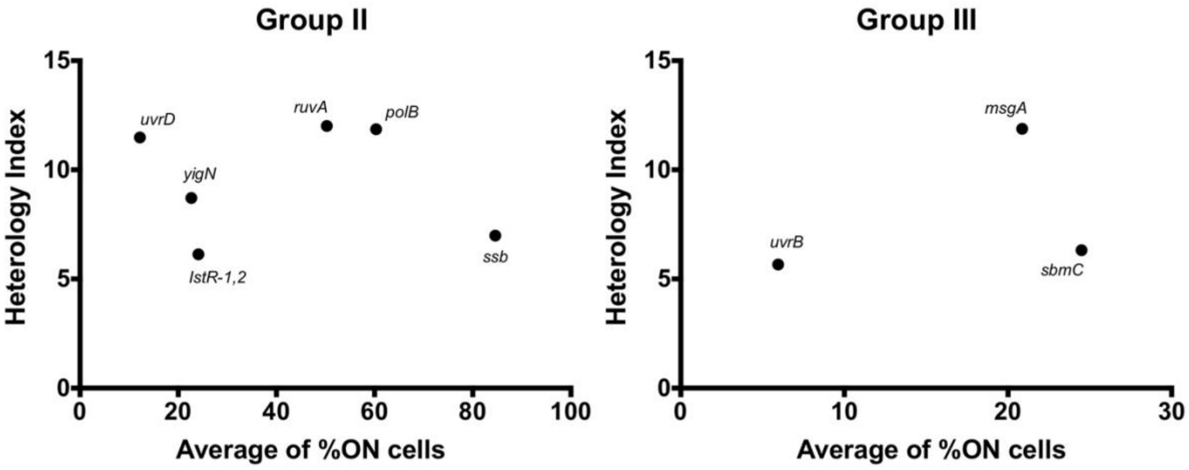

Figure S5. Correlation between the HI values of SOS boxes and the sizes of the ON subpopulations of genes classified into groups II and III.
