# Supplementary figures and images for: Genome-Wide Identification and Expression Analysis of SOS Response Genes in Salmonella enterica Serovar Typhimurium
Source: Cells. 2021 Apr 19;10(4):943. doi: 10.3390/cells10040943 (PMC8072944; doi:10.3390/cells10040943)

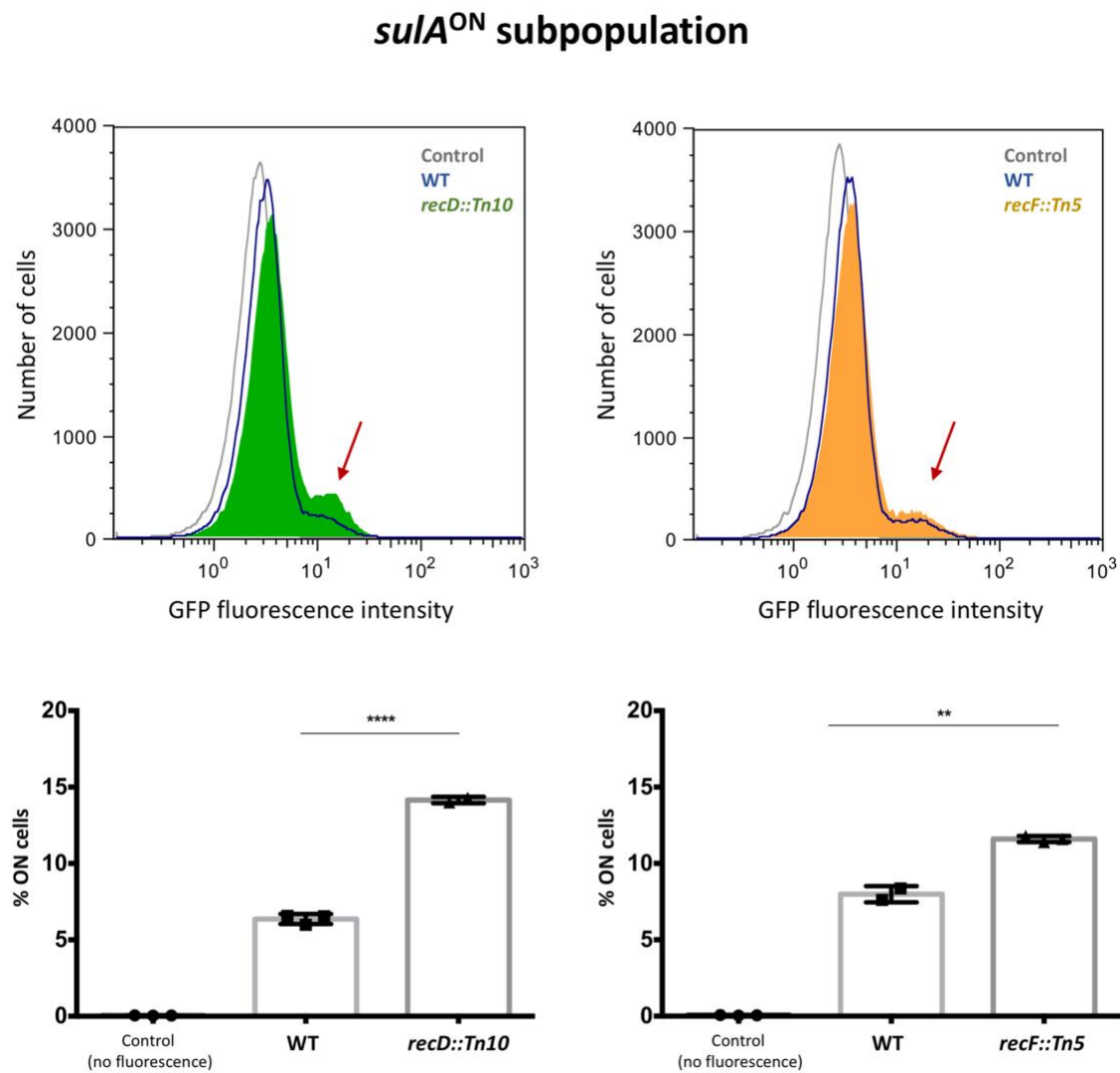

**Figure S2.** Sizes of the *SulA*<sup>ON</sup> subpopulation in *recD* and *recF* backgrounds.

Supplement: Supplementary file 1 [file cells-10-00943-s001.zip › Figure S2.pdf]
